# Long-term safety and frequency of repeat zuranolone treatment in patients with major depressive disorder rolling over from the randomised CORAL Study into the open-label SHORELINE Study

**DOI:** 10.1192/j.eurpsy.2024.678

**Published:** 2024-08-27

**Authors:** G. W. Mattingly, S. J. Mathew, S. V. Parikh, S. T. Aaronson, B. T. Baune, A. Czysz, I. Nandy, V. Ona, C. Brown, S. Kyaga, F. Forrestal, S. Levin, J. Doherty, G. Mattingly

**Affiliations:** ^1^ Washington University, St. Louis; ^2^Baylor College of Medicine, Houston; ^3^University of Michigan, Ann Arbor; ^4^Department of Psychiatry, Sheppard Pratt Health System and University of Maryland School of Medicine, Baltimore, United States; ^5^Department of Psychiatry, University of Münster, Münster, Germany; ^6^Sage Therapeutics, Inc.; ^7^Biogen Inc., Cambridge; ^8^ Washington University, St. Louis, MO, United States

## Abstract

**Introduction:**

Zuranolone (ZRN) is a positive allosteric modulator of both synaptic and extrasynaptic gamma-aminobutyric acid type A receptors and a neuroactive steroid approved as an oral, once-daily, 14-day treatment course for adults with postpartum depression in the US and under investigation for adults with major depressive disorder (MDD). The randomised, double-blind, placebo-controlled, Phase 3 CORAL Study assessed the efficacy and safety of ZRN 50 mg vs placebo, each co-initiated with an open-label standard-of-care antidepressant (ADT). Patients who completed CORAL could roll over into open-label SHORELINE, which assessed the safety and tolerability of ZRN 50 mg and need for repeat treatment courses in adults with MDD.

**Objectives:**

To assess the safety and tolerability (primary endpoint) and need for repeat ZRN 50 mg treatment courses (secondary endpoint) in adults with MDD who previously enrolled in CORAL.

**Methods:**

CORAL enrolled adults (18–64 years) with MDD and 17-item Hamilton Rating Scale for Depression (HAMD-17) total score ≥24. After completing the 6-week CORAL Study, patients who enrolled in SHORELINE could enter a 46-week observation period to assess the safety and need for 14-day repeat ZRN treatment course(s), with a total of ≤4 repeat treatment courses permitted. Patients were screened every 2 weeks with the 9-item Patient Health Questionnaire, and scores ≥10 prompted a HAMD-17 assessment within 1 week. Patients with HAMD-17 total score ≥20 were eligible for repeat ZRN course(s) ≥8 weeks after completing the prior ZRN treatment course.

**Results:**

Among the 190 patients from CORAL who rolled over into SHORELINE and received ≥1 ZRN treatment course in either study, 133 (70.0%) had received ZRN+ADT and 57 (30.0%) received placebo+ADT in CORAL. Overall, 118 rollover patients received ≥1 open-label ZRN treatment course in SHORELINE. For patients who received ≥1 ZRN treatment course in either study, 76.8% received 1 (54.2%; 103/190) or 2 (22.6%; 43/190) total ZRN treatment courses across both studies in up to 1 year in study. The most common (>5%) treatment-emergent adverse events (TEAEs) during treatment and 14 days following the last ZRN dose were somnolence (16.1% of patients), dizziness (8.5%), headache (8.5%), fatigue (7.6%), sedation (5.9%), and nausea (5.1%); study-period TEAEs (73.7%; 87/118) for the majority of patients were mild/moderate (69.5%; 82/118) in severity and occurred primarily during the treatment period (58.5%; 69/118). No signals for increased suicidal ideation/behaviour were observed.

**Conclusions:**

Safety and tolerability among rollover patients were consistent with previous studies; most of the TEAEs reported by adults with MDD who received ZRN were mild/moderate in severity. Most patients who rolled over from CORAL to SHORELINE received ≤2 total treatment courses in up to 1 year in study.

**Disclosure of Interest:**

G. Mattingly Grant / Research support from: Akili, Alkermes, Allergan (now AbbVie), Axsome, Boehringer, Janssen, Lundbeck, Medgenics, NLS-1 Pharma AG, Otsuka, Reckitt Benckiser, Roche, Sage, Sunovion, Supernus, Takeda, and Teva, Consultant of: Akili, Alkermes, Allergan (now AbbVie), Axsome, Ironshore, Intra-Cellular Therapies, Janssen, Lundbeck, Neos Therapeutics, Otsuka, Purdue, Rhodes, Sage, Sunovion, Takeda, and Teva, Speakers bureau of: Alkermes, Allergan (now AbbVie), Ironshore, Janssen, Lundbeck, Otsuka, Sunovion, and Takeda, S. Mathew Grant / Research support from: Biohaven Pharmaceuticals, Boehringer-Ingelheim, Janssen, Merck, Sage Therapeutics, Inc., and VistaGen Therapeutics, Consultant of: Allergan (now AbbVie), Alkermes, Almatica Pharma, Axsome Therapeutics, BioXcel Therapeutics, Boehringer-Ingelheim, Clexio Biosciences, COMPASS Pathways, Eleusis, EMA Wellness, Engrail Therapeutics, Greenwich Biosciences, Intra-Cellular Therapies, Janssen, Levo Therapeutics, Perception Neurosciences, Praxis Precision Medicines, Neumora, Neurocrine, Relmada Therapeutics, Sage Therapeutics, Inc., Seelos Therapeutics, Signant Health, and Sunovion, S. Parikh Grant / Research support from: Aifred, Assurex, Janssen, Mensante, Sage Therapeutics, Inc., and Takeda, S. Aaronson Grant / Research support from: COMPASS Pathways and Neuronetics; has served as a consultant to Genomind, Inc., Janssen, LivaNova PLC, Neuronetics; and Sage Therapeutics, Inc., Speakers bureau of: Janssen and Sunovion Pharmaceuticals, Inc., B. Baune Speakers bureau of: Angelini, AstraZeneca, Biogen, Bristol Myers Squibb, Janssen, LivaNova, Lundbeck, Novartis, Otsuka, Pfizer, Servier, Sumitomo Pharma, Wyeth, and Boehringer-Ingelheim, A. Czysz Shareolder of: Sage Therapeutics, Inc., Employee of: Sage Therapeutics, Inc., I. Nandy Shareolder of: Sage Therapeutics, Inc., Employee of: Sage Therapeutics, Inc., V. Ona Shareolder of: Sage Therapeutics, Inc., Employee of: Sage Therapeutics, Inc., C. Brown Shareolder of: Sage Therapeutics, Inc., Employee of: Sage Therapeutics, Inc., S. Kyaga Employee of: Biogen Inc., F. Forrestal Employee of: Biogen Inc., S. Levin Employee of: Biogen Inc., J. Doherty Shareolder of: Sage Therapeutics, Inc., Employee of: Sage Therapeutics, Inc., G. Mattingly: None Declared

